# Current practices in outpatient parenteral antimicrobial therapy programmes: an international multi-centre survey

**DOI:** 10.1093/jacamr/dlaf075

**Published:** 2025-05-27

**Authors:** Zenaw T Wolie, Jason A Roberts, Luis Eduardo López-Cortés, Manuel Mirón-Rubio, James Pollard, Afra Nahdia Marizan Nor, Mohd H Abdul-Aziz, Conor Jamieson, R Andrew Seaton, Mark Gilchrist, Fekade B Sime

**Affiliations:** Centre for Clinical Research, Faculty of Health, Medicine and Behavioural Sciences, The University of Queensland, Brisbane, QLD 4029, Australia; Department of Pharmacy, College of medicine and health Sciences, Debre Markos University, Debre Markos, Ethiopia; Centre for Clinical Research, Faculty of Health, Medicine and Behavioural Sciences, The University of Queensland, Brisbane, QLD 4029, Australia; Herston Infectious Diseases Institute (HeIDI), Metro North Health, Brisbane, QLD, Australia; Departments of Pharmacy and Intensive Care Medicine, Royal Brisbane and Women’s Hospital, Brisbane, QLD 4029, Australia; UR UM 103, University of Montpellier, Division of Anesthesia Critical Care and Emergency and Pain Medicine, Nimes University Hospital, Nimes, France; Unidad Clínica de Enfermedades Infecciosas y Microbiología, Instituto de Biomedicina de Sevilla (IBiS)/CSIC, Seville, Spain; Hospital Universitario Virgen Macarena, and Departamento de Medicina, Universidad de Sevilla, Seville, Spain; CIBERINFEC, Instituto de Salud Carlos III, Madrid, Spain; Hospital at Home Service, Hospital Universitario de Torrejón, Madrid, Spain (on behalf of the Spanish Society of Hospital at Home (SEHAD)); Cabrini @ Home, Cabrini Health, Melbourne, Australia; Pharmacy Department, Hospital Sungai Buloh, Selangor, Malaysia; Centre for Clinical Research, Faculty of Health, Medicine and Behavioural Sciences, The University of Queensland, Brisbane, QLD 4029, Australia; Medical Directorate, NHS England (Midlands), Birmingham, UK; Department of Infectious Diseases, Queen Elizabeth University Hospital, Glasgow, UK; OPAT Initiative, British Society for Antimicrobial Chemotherapy (BSAC), Birmingham, UK; OPAT Initiative, British Society for Antimicrobial Chemotherapy (BSAC), Birmingham, UK; Department of Pharmacy/Infection, Imperial College Healthcare NHS Trust, London, UK; Department of Infectious Diseases, Imperial College London, London, UK; Centre for Clinical Research, Faculty of Health, Medicine and Behavioural Sciences, The University of Queensland, Brisbane, QLD 4029, Australia

## Abstract

**Objectives:**

This study aimed to assess the current international practices of outpatient parenteral antimicrobial therapy (OPAT).

**Methods:**

A multi-site cross-sectional study was conducted, using REDCap platform for survey distribution and data collection. Data analysis was performed in SPSS version 29.

**Results:**

Of the 146 OPAT responding centres serving adults, paediatrics or both, 82% have been operating for over 10 years. OPAT teams were multidisciplinary, with nurses comprising the largest proportion in 89% of centres, followed by infectious disease (ID) pharmacists (83%) and ID physicians (80%). Service activity varied widely, with 92% centres treating fewer than 100–5000 patients annually (median = 300). Home-based models of care were used by more than 85% of facilities in Australia, the UK and Spain, while 82% of Malaysian and 75% of UK centres used infusion centres. Common OPAT indications include skin and soft tissue, bone and joint, urinary tract and respiratory tract infections, with ceftriaxone and ertapenem used by over 80% of the centres. Peripherally inserted central and midline catheters were common except in Malaysia. Among enrolled centres, complex oral antimicrobial therapy supervision was higher in the UK (82%) than in Spain (77%), Australia (42%) and Malaysia (24%). Inconsistencies in guidelines supporting antimicrobial stability and dosing information were noted, with a call for more stability data on certain antimicrobials.

**Conclusions:**

This study revealed significant variation in OPAT practice. Further study is needed to understand the reasons for these differences and whether better alignment of practice could improve patient outcomes, antimicrobial stewardship practice and resource utilization.

## Background

The use of parenteral antimicrobials in the community is increasing. This practice is referred to as community-based parenteral antimicrobial therapy, hospital-in-the-home therapy, non-inpatient anti-infective therapy and, most commonly, outpatient parenteral antimicrobial therapy (OPAT).^1,2^ It involves administering parenteral antimicrobials in at least two doses on different days in an outpatient or ambulatory setting, either through early discharge or avoiding patient hospitalization.^3,4^

OPAT has multiple benefits, including improved patient welfare and satisfaction, reduced risk of healthcare-associated infections and more cost-effective use of resources. Its growing adoption is further supported by the availability of once-daily antimicrobial agents and advancements in antimicrobial delivery technology.^1,3–7^ Given these benefits, OPAT has become an essential approach for managing infections that require administration of parenteral antimicrobials across diverse patient populations.^7–11^ Common indications include skin and soft tissue infection, osteoarticular infections, complex respiratory tract infections, drug resistant urinary tract infections, intra-abdominal infections, endocarditis and bacteraemia.^12–14^

OPAT services are delivered through various care models, including home care (nurse-led or self-administration) or facility-based settings (outpatient infusion centres or community/skilled nurse facilities).^15–18^ These models are tailored based on factors including patient preferences, healthcare systems, geography, treatment availability, drug administration technology and reliability of service facilities.^19^ Of note, many contemporary OPAT programmes are evolving into a complex outpatient antimicrobial therapy (COpAT) programme, integrating intravenous (IV)-to-oral antimicrobial switching and ongoing monitoring.^20^

Despite its advantages, OPAT faces several challenges, including line- or antimicrobial-associated complications,^21^ unplanned medical care visits^22^ and antibiotic stability issues.^23^ Additionally, significant heterogeneity in service delivery at global, national and local levels challenges the establishment of standardized OPAT programmes. Understanding practice patterns and identifying existing disparities are essential for designing adaptable systems that optimize OPAT outcomes. However, comprehensive data on contemporary OPAT practice remain limited. This study aimed to assess current international practices of OPAT programmes.

## Methods

### Study design and settings

A multi-site cross-sectional survey of OPAT delivery centres was conducted from September 2023 to April 2024 using a web-based, self-administered questionnaire across selected countries in Australasia (Australia and Malaysia) and Europe (the UK and Spain).

### Study participants

The study participants were OPAT delivery centres in the study countries. Participants were invited through professional networks, including the BSAC, the Hospital in the Home (HITH) Society of Australasia, the Spanish Society of Home Hospitalization (SEHAD) and the Malaysian National OPAT Programme. Lead OPAT practitioners from each site were contacted via email, and a single response per site was collected from a team of 3–5 OPAT practitioners, including infection specialists, OPAT nurses and antimicrobial pharmacists.

### Sample size determination

To determine a representative sample size, the target population should be known or at least estimable from existing data.^4,17,24,25^ However, for this preliminary international survey, the size of the population, which was the total number of OPAT delivery sites, was unknown, making it challenging to estimate a specific sample size. Therefore, we aimed to enrol all OPAT delivery sites in the participating countries until the responses saturated (i.e. no new centres could be accessed), by leveraging the professional networks.

### Inclusion and exclusion criteria

The survey included OPAT delivery centres serving both paediatrics and adult patients, across various modalities such as at-home care, hospital outpatient infusion centres and community infusion centres. Centres without active OPAT programmes were excluded.

### Development, validation and distribution of the survey questionnaire

The questionnaire, designed with both multiple-choice and open-ended questions allowing for additional explanations, comprised 36 items categorized into five main domains: general participant information, characteristics of OPAT practices, governance and policy-related questions, stability and dosing issues (Survey S1, available as Supplementary data at *JAC-AMR* Online). The investigators developed and refined the questions using literature, international guidelines, prior OPAT surveys and their own personal experience. The survey underwent face validity testing through a review process, focusing on language clarity, logical question flow, completeness and overall relevance to the research objectives. The survey was formatted and distributed electronically via the Research Electronic Data Capture (REDCap) platform, ensuring secure and standardized data collection. It was distributed through professional networks to the lead OPAT practitioners at each site. The survey was active for 8 months, with at least two reminder emails sent to each OPAT leader.

### Data collection, management and analysis

As described in Section 2.2, a single response was collected electronically from each site. This approach relied on self-reported data, with respondents providing information from their clinical experience rather than from verified databases or standardized records. After the survey closed, data were downloaded from REDCap and analysed using SPSS statistical software (version 29). Incomplete responses were included in the analysis only if all mandatory questions were answered. Since only one response was received from each site, the possibility of duplicate responses was eliminated. Unanswered questions were treated as missing values and excluded in the analysis. Total percentages were used to demonstrate overall response distributions.

### Ethical considerations

The study was approved by the University of Queensland’s Human Research Ethics Committee (approval project ref. #2023/HE000269). Participants were provided with a detailed information page before starting the survey, outlining the study’s purpose, the investigator contact details, and the estimated survey duration. By proceeding to complete the survey, participants indicated their informed consent to voluntary completion of the survey after reviewing this information.

## Results

### General characteristics of OPAT delivery facilities

Data were collected from 146 OPAT centres across four study countries, and the general characteristics of responding centres are summarized in Table 1. Non-OPAT personnel, such as community and district nurses, support OPAT community services in some centres, ranging from 2 centres (11%) in Australia to 14 centres (42%) in the UK (Table S1).

**Table 1.** The general characteristics of OPAT delivery facilities across the study countries (N = 146)

Study parameters		Spain	UK	Australia	Malaysia
Number of participating centres in each country		77 (53%)	33 (23%)	19 (13%)	17 (12%)
Years of OPAT service establishment	1–5	17 (22%)	5 (15%)	—	16 (94%)
	6–10	7 (9%)	10 (30%)	4 (21%)	1 (6%)
	>10	53 (69%)	18 (55%)	15 (79%)	—
OPAT team composition	ID physician^a^	71 (92%)	19 (58%)	12 (63%)	15 (88%)
	ID/clinical microbiologist	17 (22%)	25 (76%)	1 (5%)	4 (24%)
	ID specialist pharmacist^b^	40 (52%)	29 (88%)	6 (32%)	15 (88%)
	OPAT nurse	68 (88%)	32 (97%)	15 (79%)	15 (88%)
	Other OPAT team members^c^	11 (14%)	20 (61%)	16 (84%)	9 (53%)
Catchment population size for the OPAT programme	1 million–4 million	1 (1%)	8 (24%)	4 (21%)	1 (6%)
	100,000–<1 million	31 (40%)	22 (67%)	8 (42%)	4 (24%)
	50,000–<100 000	4 (5%)	—	2 (11%)	—
	1000–<50 000	1 (1%)	—	—	4 (24%)
	1–<1000	31 (40%)	—	—	3 (18%)
Average number of patients treated annually	≥10 000	—	—	—	
	5000–<10 000	—	—	2 (11%)	—
	1000–<5000	12 (16%)	3 (9%)	9 (47%)	—
	100–<1000	54 (70%)	28 (85%)	7 (37%)	
	1–<100	6 (8%)	—	—	17 (100%)

^a^In some enrolled centres, ID physicians may refer to doctors receiving training in ID, but who are not specialists in ID.

^b^In some participating centres, the pharmacists may not be ID specialist pharmacists.

^c^Other OPAT team members are presented in Table S2. OPTA, outpatient parenteral antibiotic therapy.

### Characteristics of OPAT practices

#### Patient demographics and OPAT models of care

**Figure 1.**
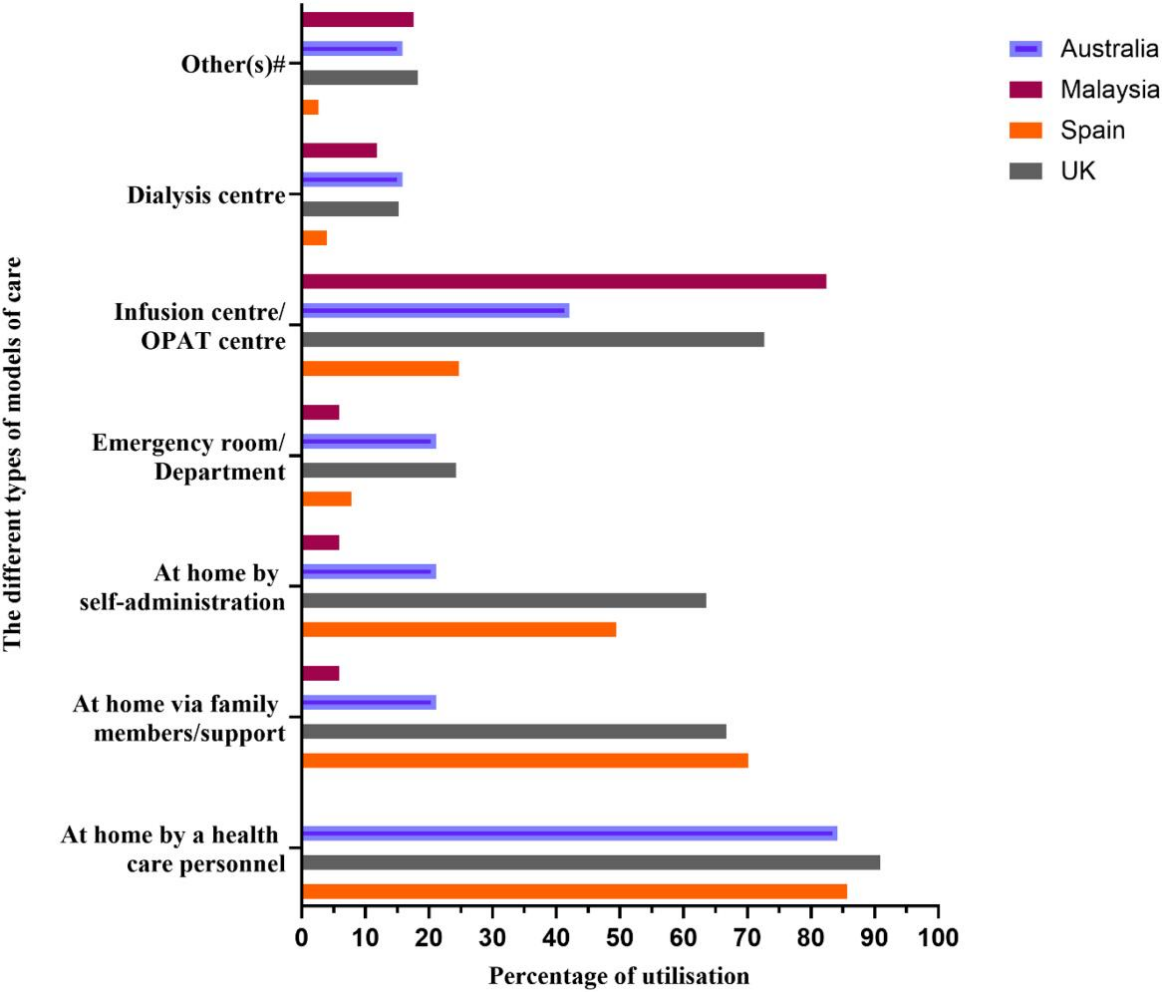
OPAT models of care and the percentage of centres reporting the use of these models (other(s)# = listed out in Table S3).

The distribution of OPAT services varied across participating countries in terms of patient demographics. The majority of enrolled centres in Malaysia (94%), the UK (88%) and Spain (68%) treated adults, while 42% of Australian centres served both adults and paediatrics (Table 2). Figure 1 illustrates the distribution of currently used OPAT care models. In Australia, the UK and Spain, over 85% of OPAT facilities provided home-based care via healthcare personnel. Home-based care with family support or self-administration was also common in the UK and Spain. In Malaysia, 82% of centres, and nearly 73% in the UK, provided services through infusion centres.

**Table 2.** OPAT-treated patient demographics and number (%) of responding centres by country

Patient demographics	Australia (*n* = 19)	Malaysia (*n* = 17)	UK (*n* = 33)	Spain (*n* = 77)
Adult	5 (26%)	16 (94%)	29 (88%)	52 (68%)
Paediatric	3 (16%)	—	2 (6%)	5 (6%)
Both	8 (42%)	1 (6%)	1 (3%)	18 (23%)

*n*, total number of enrolled centres in each study country.

#### Common indications and prescribed IV antimicrobials

Based on pooled data across all survey sites, the most common reported OPAT indications were respiratory tract infections (61.6%), urinary tract infections (59.6%), skin and soft tissue infections (53.4%), osteomyelitis (30.8%) and prosthetic joint infections (28.8%) (Table S4A) However, the frequency of these indications varies across countries, and a comprehensive summary is provided in Table S4B.

Table S5 presents antimicrobials used to treat these indications over the past 1 year in participating facilities. Most enrolled centres reported using beta-lactams, particularly ceftriaxone, ertapenem and ceftazidime, with ceftriaxone and ertapenem used in over 90% of facilities. The use of other antimicrobials, such as aminoglycosides and glycopeptides (e.g. teicoplanin and vancomycin), varies across countries. Additionally, certain antifungals, including amphotericin B and caspofungin, were used among the enrolled OPAT centres, except in Malaysia.

#### Vascular access devices and infusion pumps

**Figure 2.**
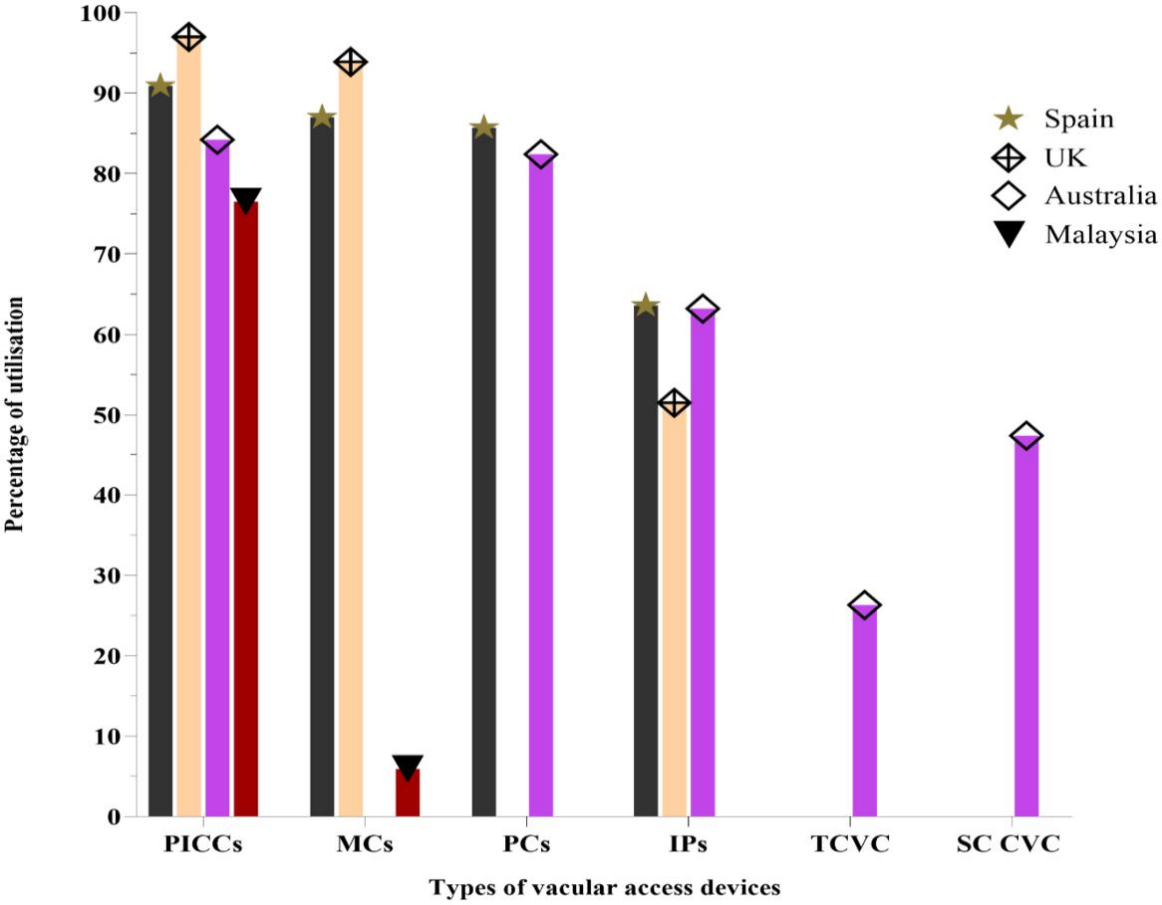
Different types of VADs and the percentage of responding centres reporting the use of these devices.

In this survey, peripherally inserted central catheters were the most commonly reported as being used by 97% of the UK, 91% of Spanish, 84% of Australian and 77% of Malaysian centres. Use of peripheral cannulas and midline catheters (MCs) was also reported among majority of enrolled centres, though the usage of MCs was low in Malaysia (6%). Overall utilization rates of various vascular access devices (VADs) are shown in Figure 2.

**Figure 3.**
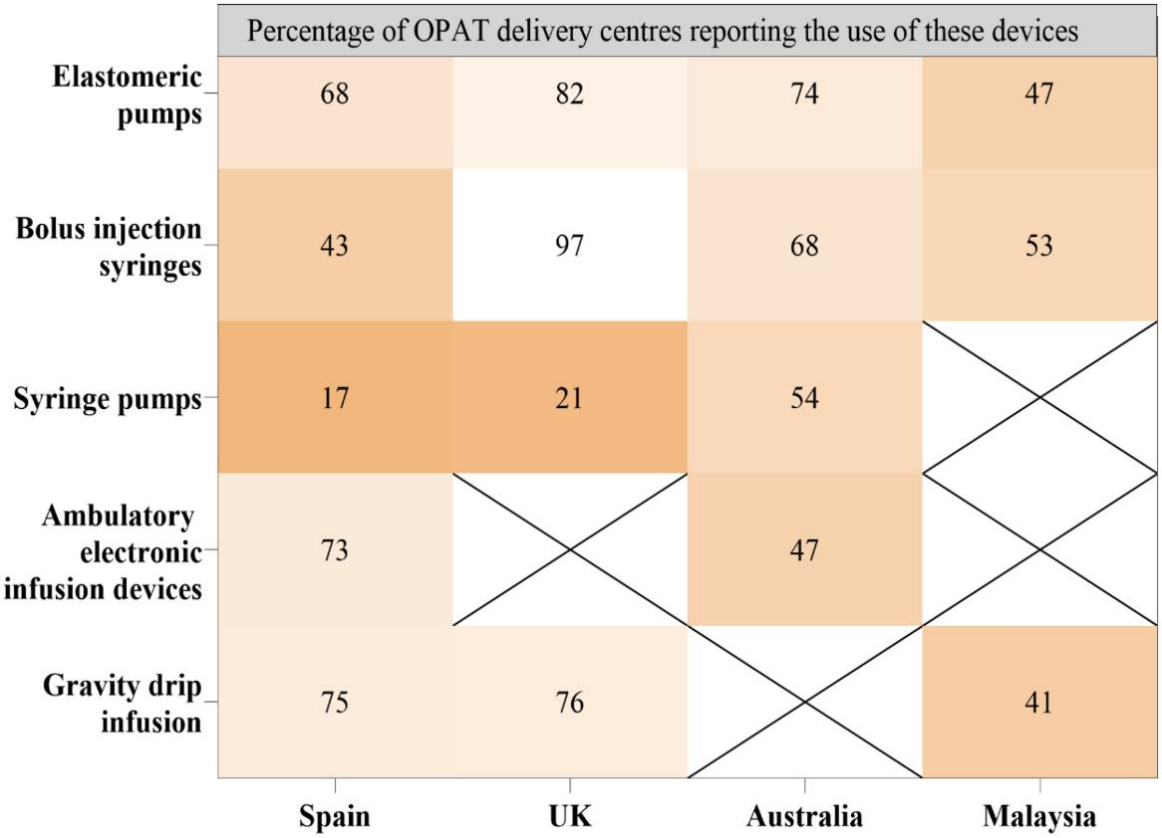
Medication administration infusion pumps and the percentage of responding centres reporting the use of these devices.

This survey also examined the use of medication administration infusion pumps in OPAT practice, revealing variations in their rate of utilization across countries (Figure 3).

#### Subcutaneous route of antimicrobial administration

In this study, 48% of the Spanish OPAT centres reported using the subcutaneous route for antimicrobial administration, compared to lower respondents in Australia (11%) and the UK (3%).

### Governance and policy-related assessment in OPAT delivery facilities

Table 3 summarizes data on OPAT services supervision, antimicrobial stewardship (AMS) activities, contributions to national registries and access to guidelines. Most OPAT facilities, ranging from 24% in Malaysia to 82% in the UK, reported supervising complex oral antimicrobial therapy. AMS activities were performed at most centres, though performance rate varies between countries. The majority of OPAT services in Australia, Malaysia and Spain are affiliated with nationally recognized healthcare models. Data contribution to national or local registries was variable, with the highest performance rate reported by the UK centres (58%).

**Table 3.** Supervision of complex oral antimicrobial therapy, antimicrobial stewardship activities and availability of guidelines across study countries

Study parameters	Response rates of OPAT centres by countries, *n* (%)			
	Spain (*n* = 77)	UK (*n* = 33)	Australia (*n* = 19)	Malaysia (*n* = 17)
Supervision of complex oral antimicrobial therapy at OPAT services	59 (77%)	27 (82%)	8 (42%)	4 (24%)
Antimicrobial stewardship activities at the OPAT centres				
Applying IV-to-PO switch criteria	56 (73%)	30 (91%)	12 (63%)	12 (71%)
Operating under antimicrobial stewardship programme	54 (70%)	30 (91%)	13 (68%)	13 (77%)
Recording of drug-related adverse events	56 (73%)	31 (94%)	14 (74%)	12 (71%)
Recording of line-related adverse events	52 (68%)	31 (94%)	15 (79%)	13 (77%)
Patient satisfaction scores	32 (42%)	24 (73%)	11 (58%)	8 (47%)
Participation in annual reports	33 (43%)	24 (73%)	9 (47%)	9 (53%)
Is the OPAT service part of a nationally recognized healthcare model?				
Yes, it is	47 (61%)	19 (58%)	13 (68%)	12 (71%)
No, it is not	16 (21%)	2 (6%)	—	1 (6%)
I don’t know	12 (16%)	10 (30%)	3 (16%)	3 (18%)
OPAT service data contribution to a national or local registry	30 (39%)	19 (58%)	7 (37%)	9 (53%)
Presence/accessibility of national clinical practice guidelines for OPAT Service	50 (65%)	30 (91%)	8 (42%)	3 (18%)
Presence/accessibility of local clinical practice guidelines for OPAT service	31 (40%)	23 (70%)	14 (74%)	13 (77%)

*n*, total number of enrolled centres in each study country; OPAT, outpatient antimicrobial therapy; IV, intravenous; PO, per oral.

Additionally, most UK centres (91%) and 65% of centres in Spain reported the availability of accessible national OPAT practice guidelines. Approximately 75% of facilities in Australia and Malaysia indicated availability of local OPAT guidelines for their clinical practice (Table S6).

### Stability data and infusion practices in OPAT service centres

#### Inclusion of stability information in guidelines

Approximately 58% of OPAT centres in Spain and the UK reported that their national guidelines included sufficient information on antimicrobial stability, compared to 42% in Australia and 24% in Malaysia. However, 53% of OPAT centres in Australia and Malaysia reported sufficient stability data availability in their local guidelines, surpassing the 46% in Spain and 21% in the UK.

#### Antimicrobials that require additional/new stability data to support use in OPAT conditions

Meropenem was the most frequently reported antimicrobial requiring additional stability data (45% of facilities), followed by ceftazidime/avibactam (32%) and meropenem/vaborbactam (25%) (Table 4). Facilities also indicated a need for more stability data for amoxicillin/clavulanic acid and ceftaroline.

**Table 4.** Antimicrobials requiring additional stability data and the use of buffered formulations in OPAT centres

Antimicrobials	OPAT delivery centres reported the need for additional/new stability data to support the use of these antimicrobials in their services, *n* (%)	OPAT delivery centres used buffered formulations of these antimicrobials to address stability concerns, *n* (%)
Aciclovir	26 (18%)	—
Amoxicillin	31 (21%)	6 (4%)
Amphotericin B	14 (10%)	—
Ampicillin	34 (23%)	—
Ampicillin/sulbactam	27 (19%)	—
Aztreonam	15 (10%)	—
Benzylpenicillin (penicillin G)	—	24 (16%)
Cefazolin	14 (10%)	—
Cefepime	19 (13%)	6 (4%)
Ceftazidime	34 (23%)	7 (5%)
Ceftazidime/avibactam	46 (32%)	—
Ceftolozane/tazobactam	29 (20%)	—
Cefiderocol	25 (17%)	—
Colistin	14 (10%)	—
Doripenem	14 (10%)	—
Flucloxacillin	—	32 (22%)
Imipenem	26 (18%)	—
Imipenem/relebactam	27 (19%)	—
Meropenem	66 (45%)	14 (10%)
Meropenem/vaborbactam	37 (25%)	—
Piperacillin/tazobactam	16 (11%)	34 (23%)
Tigecycline	16 (11%)	—
Vancomycin	17 (12%)	—

*n*, number of facilities from all participating countries.

#### Use of buffered antimicrobial formulations for OPAT administration to avoid stability concerns

Buffered antimicrobial formulations were infrequently used among participating centres, with some facilities unaware of what buffer formulations are (Table 4).

#### Acceptance limits for antimicrobial degradation and antimicrobials with limited use due to stability issues

OPAT clinicians were asked about the clinically acceptable amount of drug loss (due to degradation) over a 24 h infusion period for antimicrobials used in OPAT settings. Survey results indicated that most centres reported 5% and 10% drug loss thresholds as clinically acceptable limits (Table 5). Furthermore, a significant number of facilities (47–79%) reported that stability issues prevent the use of certain antimicrobials, primarily meropenem, amoxicillin/clavulanic acid, ceftazidime, ampicillin, aciclovir and meropenem/vaborbactam (Table S7).

**Table 5.** Clinically acceptable degradation limits of antimicrobials over a 24 h infusion period for OPAT use, with number (%) of responding centres by study country

Clinically acceptable degradation limit (%)	Number (%) of responding centres by study country			
	Australia (*n* = 19)	Malaysia (*n* = 17)	UK (*n* = 33)	Spain (*n* = 77)
1%	—	2(12)	3(9)	12 (16)
5%	2 (11)	8(47)	11 (33)	35 (45)
10%	11 (58)	4(24)	13 (39)	14 (18)
15%	1(5)	1(6)	1 (3)	4 (5)
25%	—	1(6)	1 (3)	—
Other	5 (26)	1(6)	4 (12)	12 (16)

*n*, total number of enrolled centres in each study country.

#### Sources of infusion preparations for OPAT practice

In this study, 63% of Australian and 43% of Spanish OPAT centres used both commercial and locally compounded preparations, while 77% of Malaysian centres primarily use locally compounded preparations. In the UK, 39% of centres used commercial preparations, while 21% used locally compounded preparations.

### Antimicrobial dosing information in OPAT services

#### Inclusion of antimicrobial dosing information in guidelines

**Figure 4.**
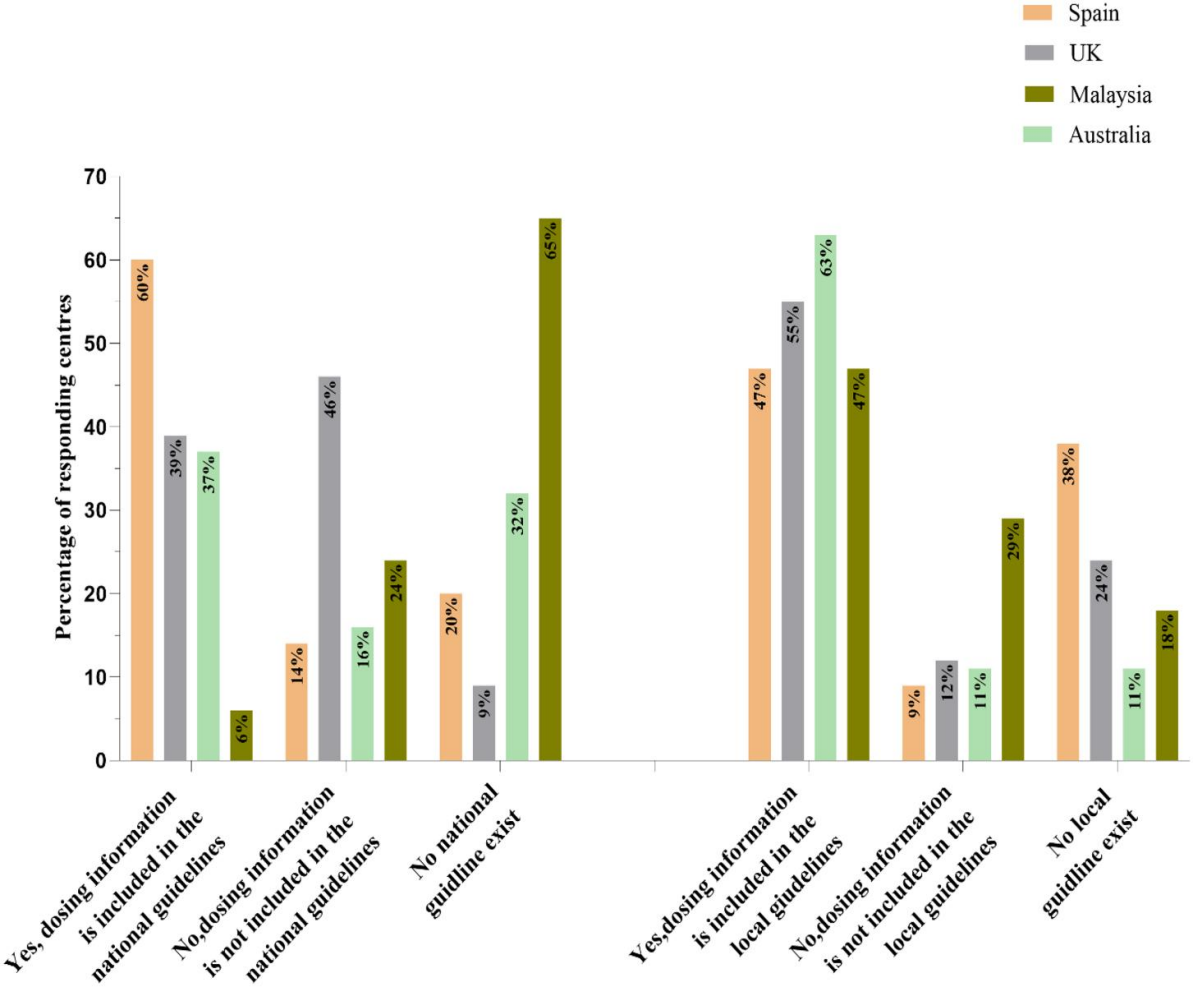
The percentage of participant facilities reporting the inclusion/absence of antimicrobial dosing information in both national and local guidelines.

Figure 4 summarizes the inclusion of sufficient antimicrobial dosing information in national and local OPAT practice guidelines. The responses highlight variations, with most centres reporting the inclusion of this data in either national or local guidelines. Notably, some indicated the absence of this information in both guideline types.

#### Twice-daily dosing of antimicrobials and bolus IV push or short infusion administration

**Figure 5.**
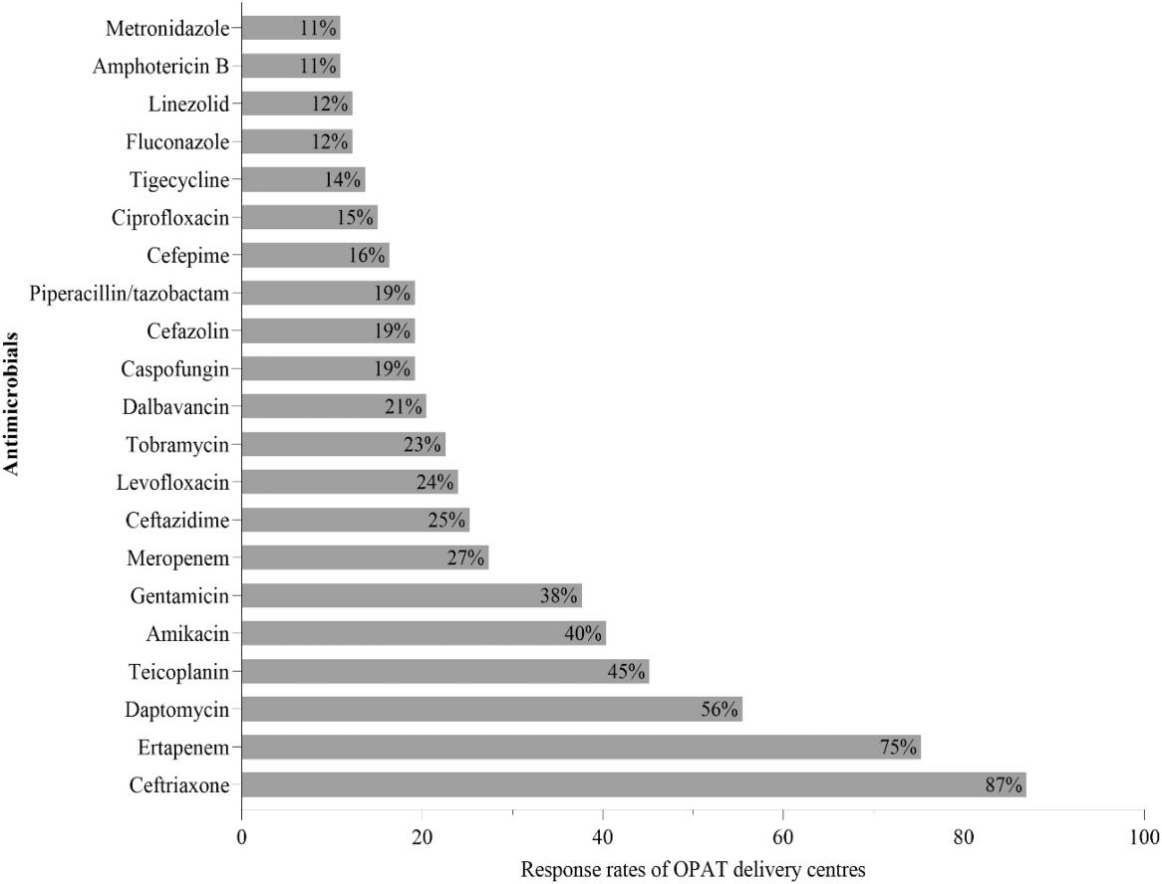
Percentage of OPAT delivery centres regarding bolus IV push or short infusion dosing of antimicrobials.

Approximately 70% of OPAT facilities reported the use of twice-daily dosing for certain antimicrobials. The most common antimicrobials administered were ceftriaxone (38%), meropenem (31%), ceftazidime (28%) and piperacillin/tazobactam (20%) (Table S8). Administration methods included either as a bolus infusion, short infusion (≤1 h) or 12 h infusion (Figure 5). Aztreonam, imipenem and vancomycin were less commonly administered this way at 10%, 10%, and 8% of centres, respectively.

#### Therapeutic drug monitoring and use of loading dose when initiating OPAT with continuous infusion

About 94% of OPAT enrolled delivery centres in the UK, 74% in Australia, 62% in Spain and 41% in Malaysia performed therapeutic drug monitoring (TDM) for antimicrobials in their OPAT settings. Table 6 shows antimicrobials routinely subjected to TDM, expressed as a percentage of responding centres relative to the total number of enrolled OPAT centres. Linezolid, fluconazole and meropenem were less commonly monitored, reported by ≤10% of the total participating centres.

**Table 6.** Antimicrobials and the number (%) of OPAT facilities performing TDM for each drug, relative to the total number of enrolled OPAT centres

Antimicrobials	Number (%) of centres reporting performance of TDM (*N* = 146)
Amikacin	59 (40%)
Vancomycin	59 (40%)
Gentamicin	49 (34%)
Teicoplanin	35 (24%)
Tobramycin	34 (23%)
Linezolid	13 (9%)
Daptomycin	12 (8%)

*N*, total number of enrolled centres across the study countries; TDM, therapeutic drug monitoring.

Additionally, approximately 48% of OPAT centres used a loading dose for some antimicrobials, including piperacillin/tazobactam, teicoplanin, ceftazidime and vancomycin, with 20%, 16%, 12% and 11% of facilities, respectively, reporting this practice. Flucloxacillin, benzylpenicillin and caspofungin were occasionally administered as loading doses during OPAT initiation with continuous infusion (CI), reported by fewer than 7% of centres.

#### Dosing regimens of ceftazidime and meropenem in OPAT

Meropenem and ceftazidime dosing regimens vary across OPAT centres (Table S9). The most common meropenem regimen (used by 33% of centres) was a 1–2 g bolus infused thrice daily, while others reported using 2 g (10%) or 3 g (8%) over 12 h and 6 g (8%) over 24 h. For ceftazidime, 25% of centres used 2 g CI every 8 h, 24% used a 6 g 24 h infusion and 11% each used 2 g or 3 g over 12 h.

## Discussion

OPAT has become an integral component of global patient care, though its development has varied across regions.^26–28^ While some countries have well-established OPAT programmes with extensive experience, others have recently begun implementing such initiatives. This variability can be attributed to factors such as healthcare infrastructure, resource availability, regulatory frameworks and local healthcare needs.

OPAT serves a wide range of populations, with service configuration determined by disease burden, patient preferences, OPAT provider expertise, care models and available resources.^3,29^ Most surveyed centres primarily treat adult populations, though this finding may reflect centre-specific response biases.

This study highlights the diversity in OPAT care models, consistent with previous findings,^30^ indicating that the availability of various care models allows healthcare professionals to customize services to effectively address patient needs.^31,32^ A multidisciplinary team is key to successful OPAT implementation, although its composition can vary based on several factors.^3,26,33^ Tailoring OPAT services based on population size, catchment area and patient load is essential for optimal resource allocation and enhancing treatment outcomes.

The clinical indications for OPAT identified in our study align with established literature,^3,34^ demonstrating its versatility in infection management. Beta-lactams were frequently prescribed, likely due to their broad-spectrum activity, efficacy, safety and ease of administration.^35,36^ Other antimicrobials, including gentamicin, teicoplanin and vancomycin, were used across centres. Given the high risk of toxicity, particularly nephrotoxicity, associated with vancomycin and gentamicin in OPAT, close patient follow-up and TDM are essential.^30^ However, the need for frequent dose adjustments and monitoring could be complex due to the labour-intensive nature of TDM for OPAT staff, assay limitations and logistical issues.^37^ A minimum of weekly TDM is recommended, with more frequent monitoring when renal function fluctuates, or dosing adjustments are needed.^37^

Our study also revealed variation in the use of both VADs and infusion devices, which could be attributed to factors such as treatment durations, patient-specific considerations, risk of complications, ease of use, efficiency logistical efficiency and therapeutic requirements.^27,38–40^ The appropriate selection of devices remains essential for accurate dosing, prevention of medication errors and optimization of AMS.

There is growing clinical evidence supporting oral therapy for complex infections, which has led to the development of COpAT. This model enables earlier transitions from parenteral to oral antimicrobials, promoting more flexible treatment options.^33,41,42^ In countries like the UK and Spain, COpAT is supervised as part of their AMS programmes. The widespread participation of OPAT centres in AMS activities demonstrates a shared commitment to improving patient outcomes and combating antimicrobial resistance. Integrating OPAT into national healthcare systems further strengthens these efforts by creating standardized frameworks for implementation and monitoring.

Standardizing OPAT practices is vital to ensuring safe, high-quality and cost-effective treatment while minimizing potential side effects.^43^ One significant challenge is the absence of universally accepted drug degradation limits. The UK’s Yellow Cover Document, supported by the BSAC OPAT guidelines, specifies a degradation limit of <5%, but our study found variability in perceived acceptable limits, even within the UK centres (mostly 5% and 10%). This suggests inconsistency in the interpretation or implementation of guidelines.

In addition, existing guidelines, such as those from the UK and the Infectious Diseases Society of America, lack specific dosing recommendations for all antimicrobial agents used in OPAT,^27,30^ potentially compromising treatment outcomes and patient satisfaction. These limitations highlight the need for integrating comprehensive antimicrobial stability data and dosing information into harmonized guidelines and ensuring their accessibility to OPAT centres, which remains crucial for effective OPAT management.^3^ Given the variability in available guidelines and the absence of such resources at some centres, developing and standardizing comprehensive national guidelines—further refined through locally adapted protocols—is essential. As OPAT continues to expand, addressing these challenges through well-designed clinical studies and establishing international or national consensus on acceptable degradation limits for 24 h OPAT infusions will be vital for the continued success and growth of OPAT.

While this international survey provides valuable insights, it has certain limitations. First, the exact number and locations of OPAT delivery centres remain unknown, making it difficult to determine appropriate sample sizes. Second, the number of centres from Australia and the UK that responded to this survey is relatively lower, potentially limiting the representativeness of the data—particularly for paediatrics OPAT—across broader geographical areas. Third, some responses may reflect centre-specific practices, rather than national OPAT delivery standards. Furthermore, data collection was reliant on respondent’s knowledge and understanding of survey questions, potentially introducing bias. For example, the estimation of catchment population sizes was based on the respondents’ knowledge rather than verified hospital records. Finally, the survey did not collect data on the relative proportions of the different OPAT care models used at each facility.

### Conclusion

OPAT is a crucial component of healthcare systems, demonstrating dynamic growth and expansion. Despite similarities in patient demographics, team compositions and care models, important variability exists in antimicrobial selection, administration devices and availability of stability and dosing information for antimicrobials used in OPAT.

Additional stability data are needed for certain antimicrobials along with optimization of dosing regimens through robust clinical studies. Standardization of OPAT practice is essential to ensure consistent treatment outcomes, enhance patient safety and improve the overall quality and cost-effective care.

## Supplementary Material

dlaf075_Supplementary_Data

## Transparency declarations

F.B.S received research grant from Pfizer 418 (2022–23), Gilead Australia Fellowship (2023) and British Society for Antimicrobial Chemotherapy, BSAC (2021–23). J.A.R. is a consultant/advisory board of Qpex (2022), Gilead (2022), Advanz Pharma (2022), Pfizer (2020), Sandoz (2020), Wolters Kluwer (2021), MSD (2021) and Summit Pharma (2021). J.A.R. received funding from Pfizer (2023), MSD (2022), Gilead (2022) and Cipla (2021) to speak at conferences. J.A.R. received industry grants from Pfizer (2023), QPEX (2021) and BSAC (2020–23). M.G. is a consultant/advisory board of Pfizer (2023) and Menarini (2023). M.G. is a co-lead of the BSAC OPAT Initiative. R.A.S. is a consultant/advisory board and/or speaker for Pfizer, Menarini, Shionogi and Napp (2023–24). R.A.S. is a co-lead of the BSAC OPAT initiative.

## Supplementary data

Survey S1 and Tables S1–S9 are available as Supplementary data at *JAC-AMR* Online.
