# Supplementary material for: Current practices in outpatient parenteral antimicrobial therapy programmes: an international multi-centre survey
Source: JAC Antimicrob Resist. 2025 May 27;7(3):dlaf075. doi: 10.1093/jacamr/dlaf075 (PMC12107060; doi:10.1093/jacamr/dlaf075)
Supplement: dlaf075_Supplementary_Data [file dlaf075_supplementary_data.zip › Supplementary file_2_Tables .docx]

**Table S1**: Non-OPAT/HITH personnel that support OPAT/HITH community services, September 2023 – April 2024.

| **Role** | Number of centres that responded to this survey questions |
| --- | --- |
| Community Nursing | 5 |
| District nurses, Hospital at Home nurses, Emergency Department teams | 2 |
| Community IV team | 1 |
| Acute clinical team | 1 |
| Emergency Service 112 at night | 1 |
| HITH SMO (Specialist Medical Officer) | 1 |
| Infectious Disease Consultant | 1 |
| Microbiologist and infectiology | 1 |
| Palliative care units, Psychiatric hospital at home unit, Pediatric Hospital at home unit | 1 |
| Palliative Care at-home providers | 1 |
| Palliative Patients Unit and At Home Primary Care Facilities | 1 |
| Pharmacy and support staff | 1 |
| Primary care nurse | 1 |
| Private provider with trained IV community nurses and Lincolnshire community health NHS IV therapy nurses | 1 |
| Psychiatric, wound dressing, post-partum care, palliative | 1 |
| Psychologist | 1 |
| Public Health Unit | 1 |

**Table S2**: OPAT team members other than ID physician, ID/clinical microbiologist, (ID specialist) pharmacist, and OPAT nurse, September 2023 – April 2024.

| Role | Number of centres that responded |
| --- | --- |
| Administrative staff | 6 |
| GP specialists | 5 |
| Medical Assistant | 4 |
| Allied Health (OT, PT, social work, podiatry, speech, neuropsychic, dieticians, car coordination) | 2 |
| Antimicrobial Pharmacy Technician | 2 |
| Emergency Physician | 2 |
| Occupational Therapist | 2 |
| Paediatrician | 2 |
| Pharmacy assistant | 2 |
| Social Work | 2 |
| Advanced Clinical Practitioner | 1 |
| Aged Care Physician | 1 |
| Clinical Pharmacist | 1 |
| Community Care Assistants | 1 |
| Community nursing team | 1 |
| Consultant endocrinologist | 1 |
| Dietetics | 1 |
| District nurses | 1 |
| Family medicine | 1 |
| Health care support worker | 1 |
| ID physician on oncology | 1 |
| Internal Medicine Physicians | 1 |
| Medical team (registrars, HMO) | 1 |
| Nurse Practitioner | 1 |
| Nursing | 1 |
| Nursing Care Technician | 1 |
| Oncology services | 1 |
| OPAT Consultant Lead (Medical consultant) | 1 |
| OPAT Data Analyst | 1 |
| Pathway Coordinator | 1 |
| Podiatrist / podiatric surgeon | 1 |
| Psychologist | 1 |
| Respiratory nurse | 1 |
| Service Managers / Trainees from Medicine & Pharmacy on rotation | 1 |
| Social Workers | 1 |
| Specialist Pharmacy Technician | 1 |
| Speech therapist | 1 |
| TPN/IV Admixture Pharmacist | 1 |
| Vascular surgeon | 1 |
| Ward Nurses | 1 |

**Table S3**: Other OPAT care models reported by enrolled delivery centres, September 2023 – April 2024.

| **List of other OPAT models** | **Number of centres that responded** |
| --- | --- |
| Daycare centre | 3 |
| CSS | 2 |
| Schools | 2 |
| Ambulatory emergency care centre, medical day care unit | 1 |
| community hospital | 1 |
| Day unit | 1 |
| Hospice | 1 |
| Hospital daycare centre | 1 |
| Local hospital | 1 |
| Nursing homes, in-patient rehab centres | 1 |
| Medical day unit hub | 1 |
| Patient's workplaces | 1 |
| Public Park/playground | 1 |
| Local accommodation | 1 |

**Table S4 A:** List of OPAT indications and overall number (%) of enrolled centres reporting these indications (N=146), September 2023 – April 2024.

| **OPAT indications** | **Number of centres reporting these indications (n)** | **Percentage (%)** |
| --- | --- | --- |
| Respiratory tract infections (including bronchiectasis) | 90 | 61.6 |
| Urinary tract infections | 87 | 59.6 |
| Skin and soft tissue infections | 78 | 53.4 |
| Osteomyelitis | 45 | 30.8 |
| Prosthetic joint infections | 42 | 28.8 |
| Intra-abdominal/pelvic infections | 37 | 25.3 |
| Infective endocarditis | 32 | 21.9 |
| Diabetic foot infection | 31 | 21.2 |
| Central nervous system infections | 15 | 10.3 |
| Other indications† | 15 | 10.3 |
| Vascular graft infections | 12 | 8.2 |
| Spinal infections | 9 | 6.2 |

**Table S4 B:** List of OPAT indications and response rates by country, September 2023 – April 2024**.**

| Indications | Number/percent (n/%) of centres reporting these indications by study country | |
| --- | --- | --- |
|  | **Australia (N=19)** | |
|  | **Number of sites (n)** | **Percentage (%)** |
| Osteomyelitis | 12 | 63.2 |
| Skin and soft tissue infections | 11 | 57.9 |
| Prosthetic joint infections | 7 | 36.8 |
| Infective endocarditis | 5 | 26.3 |
| Diabetic foot infection | 5 | 26.3 |
| Respiratory tract infections (including bronchiectasis) | 3 | 15.8 |
| Urinary tract infections | 3 | 15.8 |
| Central nervous system infections | 2 | 10.5 |
|  | **Malaysia (N=17)** | |
|  | **Number of sites (n)** | **Percentage (%)** |
| Urinary tract infections | 9 | 52.9 |
| Skin and soft tissue infections | 8 | 47.1 |
| Osteomyelitis | 6 | 35.3 |
| Respiratory tract infections (including bronchiectasis) | 5 | 29.4 |
| Infective endocarditis | 5 | 29.4 |
| Intra-abdominal/pelvic infections | 5 | 29.4 |
| Prosthetic joint infections | 3 | 17.6 |
| Diabetic foot infection | 1 | 5.9 |
| Other indications† | 4 | 23.5 |
|  | **UK (N=33)** | |
|  | **Number of sites (n)** | **Percentage (%)** |
| Respiratory tract infections (including bronchiectasis) | 17 | 51.5 |
| Skin and soft tissue infections | 21 | 63.6 |
| Prosthetic joint infections | 14 | 42.4 |
| Osteomyelitis | 13 | 39.4 |
| Diabetic foot infection | 13 | 39.4 |
| Central nervous system infections | 7 | 21.2 |
| Urinary tract infections | 7 | 21.2 |
| Intra-abdominal/pelvic infections | 7 | 21.2 |
| Spinal infections | 7 | 21.2 |
| Infective endocarditis | 8 | 24.2 |
| Vascular graft infections | 2 | 6.1 |
| Other indications† | 5 | 15.2 |
|  | **Spain (N=77)** | |
|  | **Number of sites (n)** | **Percentage (%)** |
| Urinary tract infections | 68 | 88.3 |
| Respiratory tract infections (including bronchiectasis) | 65 | 84.4 |
| Skin and soft tissue infections | 38 | 49.4 |
| Intra-abdominal/pelvic infections | 25 | 32.5 |
| Prosthetic joint infections | 18 | 23.4 |
| Osteomyelitis | 14 | 18.2 |
| Infective endocarditis | 14 | 18.2 |
| Diabetic foot infection | 12 | 15.6 |
| Vascular graft infections | 10 | 13.0 |
| Central nervous system infections | 6 | 7.8 |
| Spinal infections | 2 | 2.6 |
| Other indications† | 6 | 7.8 |

†=Bacteraemia (4), ENT infection (1), Encephalitis (1), Fever in under 3-month-old (1), Heart failure (1), Hydatid/Leishmaniasis/Syphilis (1), Liver abscess/Renal abscess (1), Malignant otitis externa (1), Melioidosis (2).

Table S5: List of antimicrobials used in the last one years in the study countries, September 2023 – April 2024.

| **Country** | **IV Antimicrobials by classes** | Number (%) of reporting centres |
| --- | --- | --- |
| **Australia (N=19)** | **Beta-Lactams and Beta-lactam beta-lactamase inhibitor combinations** |  |
|  | Benzylpenicillin (Penicillin G) | 16 (84%) |
|  | Cefazolin | 16 (84%) |
|  | Ceftriaxone | 16 (84%) |
|  | Flucloxacillin | 16 (84%) |
|  | Piperacillin/tazobactam | 16 (84%) |
|  | Cefepime | 15 (79%) |
|  | Ertapenem | 15 (79%) |
|  | Ceftazidime | 14 (74%) |
|  | Meropenem | 13 (68%) |
|  | Cephalexin | 5 (26%) |
|  | Imipenem | 5 (26%) |
|  | Ceftazidime/avibactam | 4 (21%) |
|  | Ampicillin | 3 (16%) |
|  | Ceftolozane/tazobactam | 3 (16%) |
|  | Amoxicillin | 2 (11%) |
|  | Imipenem/relebactam | 1 (5%) |
|  | **Antifungals** |  |
|  | Amphotericin B | 13 (68%) |
|  | Caspofungin | 9 (47%) |
|  | Fluconazole | 7 (37%) |
|  | Micafungin | 3 (16%) |
|  | **Antivirals** |  |
|  | Acyclovir | 7 (37%) |
|  | Ganciclovir | 4 (21%) |
|  | Valganciclovir | 1 (5%) |
|  | **Glycopeptides** |  |
|  | Vancomycin | 15 (79%) |
|  | Teicoplanin | 10 (53%) |
|  | Dalbavancin | 5 (26%) |
|  | Oritavancin | 1 (5%) |
|  | **Aminoglycosides** |  |
|  | Amikacin | 9 (47%) |
|  | Tobramycin | 9 (47%) |
|  | Gentamicin | 12 (63%) |
|  | **Lipopeptides** |  |
|  | Daptomycin | 8 (42%) |
|  | **Oxazolidinones** |  |
|  | Linezolid | 7 (37%) |
|  | **Fluoroquinolones** |  |
|  | Ciprofloxacin | 4 (21%) |
|  | Levofloxacin | 1 (5%) |
|  | **Glycylcyclines** |  |
|  | Tigecycline | 6 (32%) |
|  | **Other antimicrobials** |  |
|  | Metronidazole | 5 (26%) |
|  | Aztreonam | 3 (16%) |
|  | Trimethoprim/sulfamethoxazole | 3 (16%) |
|  | Clarithromycin | 2 (11%) |
|  | Colistin | 1 (5%) |
| **Malaysia (N=17)** | **Beta-Lactams and Beta-lactam beta-lactamase inhibitor combinations** |  |
|  | Ceftriaxone | 17 (100%) |
|  | Ertapenem | 17 (100%) |
|  | Ceftazidime | 9 (53%) |
|  | Cefazolin | 7 (41%) |
|  | Ampicillin | 1 (6%) |
|  | Benzylpenicillin (Penicillin G) | 1 (6%) |
|  | Cefepime | 1 (6%) |
|  | Meropenem | 1 (6%) |
|  | **Glycopeptides** |  |
|  | Vancomycin | 5 (29%) |
|  | **Aminoglycosides** |  |
|  | Amikacin | 6 (35%) |
| **UK (N=33)** | **Beta-Lactams and Beta-lactam beta-lactamase inhibitor combinations** |  |
|  | Ceftriaxone | 32 (97%) |
|  | Ertapenem | 31 (94%) |
|  | Meropenem | 27 (82%) |
|  | Piperacillin/tazobactam | 27 (82%) |
|  | Ceftazidime | 25 (76%) |
|  | Flucloxacillin | 24 (73%) |
|  | Benzylpenicillin (Penicillin G) | 11 (33%) |
|  | Amoxicillin | 6 (18%) |
|  | Ceftazidime/avibactam | 3 (9%) |
|  | Ceftolozane/tazobactam | 2 (6%) |
|  | Imipenem | 2 (6%) |
|  | Cefiderocol | 1 (3%) |
|  | Cefoxitin | 1 (3%) |
|  | Imipenem/relebactam | 1 (3%) |
|  | **Antifungals** |  |
|  | Caspofungin | 18 (55%) |
|  | Amphotericin B | 13 (39%) |
|  | Fluconazole | 6 (18%) |
|  | Refafungin | 1 (3%) |
|  | **Antivirals** |  |
|  | Acyclovir | 6 (18%) |
|  | Ganciclovir | 1 (3%) |
|  | Valganciclovir | 1 (3%) |
|  | **Glycopeptides** |  |
|  | Teicoplanin | 32 (97%) |
|  | Dalbavancin | 21 (64%) |
|  | Vancomycin | 3 (9%) |
|  | Oritavancin | 1 (3%) |
|  | **Aminoglycosides** |  |
|  | Amikacin | 18 (55%) |
|  | Gentamicin | 12 (36%) |
|  | Tobramycin | 8 (24%) |
|  | **Lipopeptides** |  |
|  | Daptomycin | 28 (85%) |
|  | **Oxazolidinones** |  |
|  | Linezolid | 9 (27%) |
|  | **Fluoroquinolones** |  |
|  | Ciprofloxacin | 6 (18%) |
|  | Levofloxacin | 4 (12%) |
|  | **Glycylcyclines** |  |
|  | Tigecycline | 16 (49%) |
|  | **Other antimicrobials** |  |
|  | Aztreonam | 7 (21%) |
|  | Metronidazole | 7 (21%) |
|  | Trimethoprim/sulfamethoxazole | 5 (15%) |
|  | Clarithromycin | 2 (6%) |
|  | Colistin | 2 (6%) |
| Spain (N=77) | **Beta-Lactams and Beta-lactam beta-lactamase inhibitor combinations** |  |
|  | Ceftriaxone | 72 (94%) |
|  | Ertapenem | 72 (94%) |
|  | Piperacillin/tazobactam | 68 (88%) |
|  | Ceftazidime | 63 (82%) |
|  | Cefepime | 58 (75%) |
|  | Meropenem | 58 (75%) |
|  | Ceftazidime/avibactam | 48 (62%) |
|  | Ceftolozane/tazobactam | 48 (62%) |
|  | Ampicillin | 45 (58%) |
|  | Cefazolin | 44 (57%) |
|  | Imipenem | 28 (36%) |
|  | Benzylpenicillin (Penicillin G) | 28 (36%) |
|  | Cefoxitin | 16 (21%) |
|  | Amoxicillin | 14 (18%) |
|  | Oxacillin | 13 (17%) |
|  | Cefiderocol | 11 (14%) |
|  | Ampicillin/Sulbactam | 7 (9%) |
|  | Flucloxacillin | 5 (7%) |
|  | Meropenem/vaborbactam | 4 (5%) |
|  | Imipenem/relebactam | 3 (4%) |
|  | Cefpodoxime | 1 (1%) |
|  | **Glycopeptides** |  |
|  | Teicoplanin | 46 (60%) |
|  | Vancomycin | 45 (58%) |
|  | Dalbavancin | 42 (55%) |
|  | Oritavancin | 3 (4%) |
|  | **Aminoglycosides** |  |
|  | Amikacin | 62 (81%) |
|  | Gentamicin | 50 (65%) |
|  | Tobramycin | 38 (49%) |
|  | **Antifungals** |  |
|  | Caspofungin | 43 (56%) |
|  | Fluconazole | 39 (51%) |
|  | Amphotericin B | 38 (49%) |
|  | Micafungin | 13 (17%) |
|  | **Antivirals** |  |
|  | Acyclovir | 42 (55%) |
|  | Ganciclovir | 33 (43%) |
|  | **Lipopeptides** |  |
|  | Daptomycin | 61 (79%) |
|  | **Oxazolidinones** |  |
|  | Linezolid | 40 (52%) |
|  | **Fluoroquinolones** |  |
|  | Levofloxacin | 44 (57%) |
|  | Ciprofloxacin | 35 (46%) |
|  | **Glycylcyclines** |  |
|  | Tigecycline | 35 (46%) |
|  | **Other antimicrobials** |  |
|  | Aztreonam | 42 (55%) |
|  | Metronidazole | 31 (40%) |
|  | Colistin | 21 (27%) |
|  | Trimethoprim/sulfamethoxazole | 19 (25%) |
|  | Clarithromycin | 6 (8%) |

N=Total number of enrolled centres

Table S6: National and local guidelines accessed by countries participating in this study, September 2023 – April 2024.

| **Countries** | **Notional guidelines** | **Local guidelines** |
| --- | --- | --- |
| Australia | - Australasia institute of digital health - Adult and Paediatric Hospital in the Home Guideline (Ministry of Health) - AIDH, HITH Society Position Statement, - NSW Health HITH Guideline - Queensland Hospital in the Home Guideline - Clinical Excellence Queensland - Australian Therapeutic Guidelines - Therapeutic Guidelines online | - CHS HITH Referral, Admission, and Discharge (Adults and Children) Procedure, NSW HITH Guideline - CHS and NCH HITH Guidelines - CCLHD HITH Guidelines - Antimicrobial Home Treatment - Hospital in the Home, NSLHD - Queensland Children's Hospital OPAT Guideline - Empiric Antibiotics for Children, GCUH, CHQ at Home Antimicrobials - Victorian Department of Health HITH Guidelines - Safer Care Victoria, Grampians Health Services CPG/CPP - Cellulitis, Pneumonia, Pyelonephritis, and Infected Surgical Wound Site Eastern Health Specific Guidelines - Hospital Direct Admissions Guideline with Recommendations on Treatment Duration per Condition |
| Malaysia | - National Antibiotic Guidelines | - The OPAT Guideline Published by the Hospitals - National OPAT Guidelines - HTAR Outpatient Parenteral Antimicrobial Therapy (OPAT) Protocol 2020 - Outpatient Parenteral Antimicrobial Therapy Protocol, Hospital Kuala Lumpur 2022 - OPAT Protocol, Hospital Melaka - OPAT HTJS - Hospital Sungai Buloh OPAT Guideline - KKM OPAT Guidelines - Hospital Putrajaya OPAT Guideline |
| Spain | - Protocols of the Semi Outpatient Parenteral Antimicrobial Therapy (OPAT) - Sociedad Española de Hospitalización a Domicilio (SEHAD) - Up to Date - Outpatient Parenteral Antimicrobial Therapy (OPAT), SANDFORD - Outpatient Parenteral Antimicrobial Therapy (OPAT) by the Spanish Society of Internal Medicine - Infectious Diseases Protocol, CHUS - Vascular Access Guide of Osakidetza - Spanish Society of Infectious Diseases and Clinical Microbiology (SEIMC) OPAT Program - Spanish Society of Hospital Pharmacy (SEFH) - Outpatient Parenteral Antimicrobial Therapy from the Spanish Society of Home Hospitalization, Spain, SEIMC - National Outpatient Parenteral Antimicrobial Therapy Program - Executive Summary of Outpatient Parenteral Antimicrobial Therapy - Guidelines of the Spanish Society of Clinical Microbiology and Infectious Diseases and the Spanish Society of Home Hospitalization - Several Quality Standards Promoted by the Spanish Society of Home Hospitalization - Program for the Optimization of Antibiotic Therapy (PROA) - Spanish Society of Internal Medicine and Spanish Society of Home Hospitalization Guidelines | - Recommendations for Antibiotic Therapy Optimization (PROA) - GRP, Practical Recommendations Guide - Infectious Diseases Protocol, CHUS - PROA Salud - Pharmacy consulted guide - Local access guidelines - Local pharmacy guide - Outpatient Parenteral Antimicrobial Therapy (OPAT) Protocol, Hospital Cabueñes - Guide of the Spanish Society of Infectious Diseases and Clinical Microbiology (SEIMC) and the Spanish Society of Home Hospitalization (SEHAD) - Specific hospital pharmacy guidelines - Local hospital clínic barcelona guidelines - PROA Hospital Universitari Germans Trias i Pujol - Specific hospital/unit protocol - Protocols for Management of Infectious Diseases, Transfusions of Hemoderivatives, Management of Mild Thromboembolic Disease - Antimicrobial Guide of Hospital Universitario de Navarra |
| UK | - British Society for Antimicrobial Chemotherapy (BSAC) guideline - Updated Good Practice Recommendations for Outpatient Parenteral Antimicrobial Therapy (OPAT) in Adults and Children in the UK - Scottish Antimicrobial Prescribing Group (SAPG) | - Local OPAT Policy guideline - Local guidance based on national guidance fort trust (G1264) - Trust OPAT Guidelines - Patient suitability criteria, OPAT SOP, Shared Care Document for Community and OPAT - Local Infectious Disease Guidance, Clinical Pathways, Drug Monographs, Community Administration Policies, Trust Antimicrobial Guide - OPAT Administration Guidelines - CIVAS IV Clinical Guidelines - Antimicrobial Stewardship Clinical Guidelines - AMT (Antimicrobial Therapy) - Scottish Antimicrobial Prescribing Group (SAPG) - Our UCLH (University College London Hospitals) Guidelines - Locally Written SOP and Policy - BNSSG-Wide Local Health Authority; Medusa - Local Antimicrobial Guidelines - Local OPAT Operations Policy - Trust Antibiotic Guidelines and Stewardship Principles - Swansea Bay University Health Board |

**Table S7**: List of antimicrobials that enrolled OPAT delivery centres wish to use but cannot, due to stability issues as reported, September 2023 – April 2024.

| **Antimicrobials** | **Number of responding OPAT centres (N=146)** |
| --- | --- |
| Meropenem | 40 |
| Amoxicillin/Clavulanic Acid | 18 |
| Amoxicillin | 12 |
| Ceftazidime | 10 |
| Ampicillin | 7 |
| Aciclovir | 6 |
| Imipenem | 6 |
| Meropenem/Vaborbactam | 6 |
| Ceftazidime/Avibactam | 5 |
| Cefepime | 4 |
| Benzylpenicillin | 3 |
| Ceftolozane/Tazobactam | 3 |
| Vancomycin | 3 |
| Cefiderocol | 2 |
| Co-Amoxiclav | 2 |
| Imipenem/Relebactam | 2 |
| Piperacillin/Tazobactam | 2 |
| Ceftriaxone | 1 |
| Daptomycin | 1 |
| Ampicillin/Clavulanic Acid | 1 |
| Ampicillin/Sulbactam | 1 |
| Anidulafungin | 1 |
| Augmentin | 1 |
| Cefazolin | 1 |
| Cefotaxime | 1 |
| Cefoxitin | 1 |
| Ceftaroline | 1 |
| Ceftobiprole | 1 |
| Ceftolozane/Avibactam | 1 |
| Cloxacillin | 1 |
| Cotrimoxazole | 1 |
| Dalbavancin/Oritavancin | 1 |
| Delafloxacin | 1 |
| Fosfomycin | 1 |
| Omadacycline | 1 |
| Remdesivir | 1 |
| Teicoplanin | 1 |
| Tigecycline | 1 |

Table S8: Antimicrobials administered twice daily in OPAT practice, September 2023 – April 2024

| IV Antimicrobials | **Number (%) of responding OPAT centres (N=146)** | |
| --- | --- | --- |
|  | n | % |
| Ceftriaxone | 55 | 37.70 |
| Meropenem | 45 | 30.80 |
| Ceftazidime | 41 | 28.10 |
| Piperacillin/tazobactam | 29 | 19.90 |
| Cefepime | 28 | 19.20 |
| Ciprofloxacin | 28 | 19.20 |
| Tigecycline | 25 | 17.10 |
| Cefazolin | 18 | 12.30 |
| Vancomycin | 18 | 12.30 |
| Ceftazidime/avibactam | 17 | 11.60 |
| Levofloxacin | 15 | 10.30 |
| Linezolid | 15 | 10.30 |
| Aztreonam | 12 | 8.20 |
| Ertapenem | 11 | 7.50 |
| Teicoplanin | 10 | 6.80 |
| Ceftolozane/tazobactam | 9 | 6.20 |
| Imipenem | 9 | 6.20 |
| Amoxicillin | 8 | 5.50 |
| Ganciclovir | 8 | 5.50 |
| Ampicillin | 7 | 4.80 |
| Benzylpenicillin (Penicillin G) | 7 | 4.80 |
| Cefoxitin) | 7 | 4.80 |
| Acyclovir | 6 | 4.10 |
| Metronidazole | 6 | 4.10 |
| Trimethoprim/sulfamethoxazole | 6 | 4.10 |
| Gentamicin | 5 | 3.40 |
| Tobramycin | 5 | 3.40 |
| Amikacin | 4 | 2.70 |
| Ampicillin/Sulbactam) | 3 | 2.10 |
| Colistin | 3 | 2.10 |
| Meropenem/vaborbactam | 3 | 2.10 |
| Cefiderocol | 2 | 1.40 |
| Clarithromycin | 2 | 1.40 |
| Daptomycin | 2 | 1.40 |
| Flucloxacillin | 2 | 1.40 |
| Oxacillin | 2 | 1.40 |
| Dalbavancin | 1 | 0.70 |
| Clindamycin | 1 | 0.70 |
| Meropenem in renal impairment | 1 | 0.70 |
| Moxifloxacin | 1 | 0.70 |
| temocillin | 1 | 0.70 |

Table S9: Reported alternative dosing regimens for Meropenem and Ceftazidime used in OPAT delivery centers in the study countries, September 2023 – April 2024.

| **Meropenem** | **Ceftazidime** |
| --- | --- |
| 1 or 2 g bolus infusion, three times daily (33% of the facilities)-Most common dosing regimen | 2 g continuous infusion every 8 hours (25% of the facilities)-Most common dosing regimen |
| 2 g 12-hour infusion (10% of the facilities) | 6 g 24-hour infusion (24% of the facilities) |
| 3 g 12-hour infusion (8% of the facilities) | 2 g 12-hour infusion (11% of the facilities) |
| 6 g 24-hour infusion (8% of the facilities) | 3 g 12-hour infusion (11% of the facilities) |
| 4 g 24-hour infusion (3%) of the facilities | 2 g 24-hour infusion (4% of the facilities) |
| **Other alternative dosing regimens** | |
| 2g over 12-hour infusion + 2g bolus push | 100mg/kg/dose 8 hourly over 30minutes (maximum 4gram/dose) (Paediatric cystic fibrosis dosing) |
| 1-2g twice (BD) or three times daily (TDS) |  |
| 1-2g bolus infusion, followed by ambulatory pump for next two doses every 8 hours (TDS) with two daily visits | 100mg/kg over8 hours |
| 1.5g infused over 12 hours, twice daily | 1.5g 12- hourly infusion BD |
| 3g administered over 24 hours | 2g 8-hour infusion 3x daily, 1 dose via elsomeric pump over 8 hours. |
| 20-40 mg/kg, TDS | 2g 30min infusion TDS |
| 1g bolus injection, BD | 50mg/kg TDS or 75mg/kg BD |
| 1g every 8 hours with elastomer (100 ml/h) | 6g every 8 hours, administered over 1 hour |
| 1g every 8 hours in elastomer pump | 2g infused over one hour every 8 hours |
| 2g every 8 hours or extended over 3-hour infusion | 8g 24 hours infusion |
| 1-2g every 8 hours over a 1-hour infusion | 2g QID |
| 2g short infusion, twice daily | 8g 24hrs infusion |
| 1g bolus infusion, once daily for renal patients | 2g 3 x daily bolus |
| 20 mg/kg every 8 hours | 2 gr after dialysis |
| 1g/8h or 2g/8h as a 4-hour extended infusion; 3g over 24-hour infusion | 100-150 mg/Kg/day (3 doses) |
| 1g over 8 hours, 2g short infusion every 8 hours, or 3g over 24 hours | 8g/24h infusion or 2g/6h extended infusion |
| 3g infused over 24 hours | 1g every 8hr and 2g every 12h (no continuous infusion) |
| 2g every 8 hours | 1-2 g 1 hour infusion thrice daily (electronic pumps) |
| 2g every 8 hours | 2g short infusion TDS |
| 1g in a 1-hour infusion, TDS | 2g TDS |
| 40 mg/kg every 8 hours (Paediatrics dose) | 2g short infusion TDS |
| 3g every 24 hours as a continuous infusion | 2g TDS or BD as a bolus |
| 6g infused over 48 hours | 2 g bolus injection TDS |
| 3g IV once daily (OD) |  |
| 1g twice daily for renal impairment |  |
| 2g or 3g BD |  |
