# Supplementary material for: Current practices in outpatient parenteral antimicrobial therapy programmes: an international multi-centre survey
Source: JAC Antimicrob Resist. 2025 May 27;7(3):dlaf075. doi: 10.1093/jacamr/dlaf075 (PMC12107060; doi:10.1093/jacamr/dlaf075)
Supplement: dlaf075_Supplementary_Data [file dlaf075_supplementary_data.zip › Suplementary file_1_Survey.docx]

# **Supplementary file 1: Data collection Tool**

**These data collection questionnaires have been created using the REDCap online designer platform and will be hosted at UQCCR, UQ, once ethics approval is obtained.**

1. **General information**
2. *Country Name: Click or tap here to enter text.
3. *State or Region: Click or tap here to enter text.
4. Postcode/postal code: Click or tap here to enter text.
5. *Email address: Click or tap here to enter text.
6. *Name of your hospital/healthcare institution Click or tap here to enter text.
7. *When was your OPAT service established? ☐ 1-5 years ago ☐ 6-10 years ago ☐ >10 years ago
8. *Your OPAT team consists of (Tick all that apply)

☐ ID physician

☐ ID/clinical microbiologist

☐ ID specialist pharmacist

☐ OPAT nurse

☐ Other Click or tap here to enter text.

1. *In your center, is community service delivery supported by other non-OPAT/HITH personnels?

☐ Yes ☐ No

*If your answer is yes, please list the non-OPAT personal team members.Click or tap here to enter text.

1. *Approximately how large is the catchment population served by your OPAT program?Click or tap here to enter text.
2. *On the average, how many patients receive OPAT services at your centre annually?Click or tap here to enter text.

1. **Characteristics of OPAT Practice**
2. *You provide OPAT service for:

☐ Paediatrics.

☐ Adults

☐ Both Paediatrics and adults

☐ Other. Click or tap here to enter text.*

1. *Where do your patients receive OPAT administration? select all that apply.

☐ At home by self-administration

☐ At home via family members/support

☐ At home by a health care personnel

☐ Infusion centre /OPAT center

☐ Emergency Room / Department

☐ Dialysis centre

☐ Other (state)

1. *Select the top three most frequent indications for OPAT at your service.

☐ Infective endocarditis

☐ Skin and soft tissue infections

☐ Prosthetic joint infections

☐ Osteomyelitis

☐ Respiratory tract infections (including bronchiectasis)

☐ Central nervous system infections

☐ Urinary tract infections

☐ Intra-abdominal /pelvic infections

☐ Diabetic foot infection

☐ Spinal infections

☐ Vascular graft infections

☐ Other Click or tap here to enter text.

1. *Select (tick) all IV antimicrobials you are using for OPAT services in the last one year.

☐ Acyclovir

☐ Amikacin

☐ Amphotericin B

☐ Amoxicillin

☐ Ampicillin

☐ Ampicillin/Sulbactam

☐ Aztreonam

☐ Benzylpenicillin (Penicillin G)

☐ Caspofungin

☐ Cefazolin

☐ Cefiderocol

☐ Cefepime

☐ Cefoxitin

☐ Cefpodoxime

☐ Ceftazidime

☐ Ceftazidime/avibactam

☐ Ceftolozane/tazobactam

☐ Ceftriaxone

☐ Cephalexin

☐ Ciprofloxacin

☐ Clarithromycin

☐ Colistin

☐ Dalbavancin

☐ Daptomycin

☐ Doripenem

☐ Eravacycline

☐ Ertapenem

☐ Flucloxacillin

☐ Fluconazole

☐ Ganciclovir

☐ Gentamicin

☐ Imipenem

☐ Imipenem/relebactam

☐ Levofloxacin

☐ Linezolid

☐ Meropenem

☐ Meropenem/vaborbactam

☐ Metronidazole

☐ Micafungin

☐ Oritavancin

☐ Oxacillin

☐ Benzylpenicillin (Penicillin G)

☐ Piperacillin/tazobactam

☐ Refafungin

☐ Telavancin

☐ Teicoplanin

☐ Tigecycline

☐ Tobramycin

☐ Trimethoprim/sulfamethoxazole

☐ Valganciclovir

☐ Vancomycin

☐ Other Click or tap here to enter text.

1. *What type(s) of vascular access device is/are used in your practice? Please select (tick) all that apply.

☐ Peripherally inserted central catheter (PICC)

☐ Implantable port

☐ Midline catheters

☐ Peripheral cannula

☐ Tunnelled central venous catheter

☐ Subclavian CVC line

☐ Other Click or tap here to enter text.

1. *In your OPAT service, do you use the subcutaneous route of administration for any antimicrobials?

☐ Yes ☐ No

1. *Which type of antimicrobial drug administration technology do you use in your OPAT practice? Please select all that apply.

☐ Ambulatory electronic infusion devices

☐  Elastomeric pumps for continuous infusion (over 24 hrs devices)

☐ Elastomeric pumps for short infusion

☐ Syringe pumps

☐ Gravity drip infusion

☐ Bolus injection syringe

☐ Other Click or tap here to enter text.

1. **Governance and policy related questions**
2. *Do you supervise complex oral antimicrobial therapy in your OPAT/ HITH service?

☐ Yes ☐ No

1. *Which of the following antimicrobial stewardship activities are applicable to your OPAT centre? Tick all that apply.

☐ Applying IV-to-PO switch criteria

☐ Operating under Antimicrobial Stewardship program,

☐ Recording of drug related adverse events

☐ Recording of line related adverse events

☐ Patient satisfaction scores

☐ Participation in annual reports

1. *Is your OPAT service part of a national recognised healthcare model?

☐ Yes, it is ☐ No, it is not ☐ I do not know

1. * Does your OPAT service contribute data to a national or local Registry? ☐ Yes ☐ No
2. *Are there national clinical practice guidelines for OPAT services that you access?

☐ Yes ☐ No

If yes, please state the guidelines you access Click or tap here to enter text.

1. *Are there any local clinical practice guidelines for OPAT service that you access?

☐Yes ☐No

If yes, please state the guidelines you access Click or tap here to enter text.

1. **Antimicrobial stability**
2. *If national guidelines for OPAT services exist, is sufficient information included on the stability of antibiotics for prolonged infusions.

☐Yes, stability information is included

☐No, stability information is not included

☐ No national guidelines exist

1. *If local guidelines for OPAT services exist, is sufficient information on stability of antibiotics included?

☐Yes, stability information is included

☐No, stability information is not included

☐ No local guidelines exist

1. *Select (tick) all antimicrobials you would think require additional or new stability data to support use in OPAT?

☐ Acyclovir

☐ Amikacin

☐ Amphotericin B

☐ Amoxicillin

☐ Ampicillin

☐ Ampicillin/Sulbactam

☐ Aztreonam

☐ Benzylpenicillin (Penicillin G)

☐Caspofungin

☐ Cefazolin

☐ Cefiderocol

☐ Cefepime

☐ Cefoxitin

☐ Cefpodoxime

☐ Ceftazidime

☐ Ceftazidime/avibactam

☐ Ceftolozane/tazobactam

☐ Ceftriaxone

☐ Cephalexin

☐ Ciprofloxacin

☐ Clarithromycin

☐ Colistin

☐ Dalbavancin

☐ Daptomycin

☐ Doripenem

☐ Ertapenem

☐ Eravacycline

☐ Flucloxacillin

☐ Fluconazole

☐ Ganciclovir

☐ Gentamicin

☐ Imipenem

☐ Imipenem/relebactam

☐ Levofloxacin

☐ Linezolid

☐ Meropenem

☐ Meropenem/vaborbactam

☐ Metronidazole

☐ Micafungin

☐ Oritavancin

☐ Oxacillin

☐ Benzylpenicillin (Penicillin G)

☐ Piperacillin/tazobactam

☐ Refafungin

☐ Telavancin

☐ Teicoplanin

☐ Tigecycline

☐ Tobramycin

☐ Trimethoprim/sulfamethoxazole

☐ Valganciclovir

☐ Vancomycin

☐ Other Click or tap here to enter text.

☐ I don’t know

1. *For which of the following antimicrobials do you use buffered formulation for OPAT administration to avoid stability concerns? Select all that apply.

☐ Acyclovir

☐ Amikacin

☐ Amphotericin B

☐ Amoxicillin

☐ Ampicillin

☐ Ampicillin/Sulbactam

☐ Aztreonam

☐ Benzylpenicillin (Penicillin G)

☐ Caspofungin

☐ Cefazolin

☐ Cefiderocol

☐ Cefepime

☐ Cefoxitin

☐ Cefpodoxime

☐ Ceftazidime

☐ Ceftazidime/avibactam

☐ Ceftolozane/tazobactam

☐ Ceftriaxone

☐ Cephalexin

☐ Ciprofloxacin

☐ Clarithromycin

☐ Colistin

☐ Dalbavancin

☐ Daptomycin

☐ Doripenem

☐ Ertapenem

☐ Eravacycline

☐ Flucloxacillin

☐ Fluconazole

☐ Ganciclovir

☐ Gentamicin

☐ Imipenem

☐ Imipenem/relebactam

☐ Levofloxacin

☐ Linezolid

☐ Meropenem

☐ Meropenem/vaborbactam

☐ Metronidazole

☐ Micafungin

☐ Oritavancin

☐ Oxacillin

☐ Benzylpenicillin (Penicillin G)

☐ Piperacillin/tazobactam

☐ Refafungin

☐ Telavancin

☐ Teicoplanin

☐ Tigecycline

☐ Tobramycin

☐ Trimethoprim/sulfamethoxazole

☐ Valganciclovir

☐ Vancomycin

☐ *Other Click or tap here to enter text.

1. *In your opinion, what is the clinically acceptable amount of drug loss (due to degradation) over a 24-hour infusion period to allow use of an antimicrobial agent in an OPAT setting?

☐1% loss

☐ 5% loss

☐10% loss

☐15% loss

☐25% loss

☐ Other (comment) Click or tap here to enter text. *

1. *Are there any antimicrobials you would like to use in your OPAT practice, however cannot use currently due to lack of stability data or dosing guidelines?

☐*Yes please list: Click or tap here to enter text. ☐ No

1. *In your OPAT practice, do you use

☐ Infusion preparations from a commercial provider or

☐ Infusion preparation locally compounded at your institution (Pharmacy/Pharmacology Department)

☐ Both

1. **Dosing related questions**
2. If national guidelines for OPAT services exist, is sufficient information included on the dosing of antibiotics in OPAT setting?

☐Yes, dosing information is included

☐No, dosing information is not included

☐ No national guidelines exist

1. If local guidelines for OPAT services exist, is sufficient information on dosing of antibiotics included?

☐Yes, dosing information is included

☐No, dosing information is not included

☐ No local guidelines exist

1. *Do you use twice daily dosing in your OPAT practice for any of the antimicrobials (This may be a 12-hour infusion, bolus infusion, or short (30-minute) infusion)?

☐Yes ☐No

If yes, please identify for which antimicrobial agent twice daily dosing is considered in your OPAT practice. Tick all that apply. *

☐ Acyclovir

☐ Amikacin

☐ Amphotericin B

☐ Amoxicillin

☐ Ampicillin

☐ Ampicillin/Sulbactam

☐ Aztreonam

☐ Benzylpenicillin (Penicillin G)

☐ Caspofungin

☐ Cefazolin

☐ Cefiderocol

☐ Cefepime

☐ Cefoxitin

☐ Cefpodoxime

☐ Ceftazidime

☐ Ceftazidime/avibactam

☐ Ceftolozane/tazobactam

☐ Ceftriaxone

☐ Cephalexin

☐ Ciprofloxacin

☐ Clarithromycin

☐ Colistin

☐ Dalbavancin

☐ Daptomycin

☐ Doripenem

☐ Ertapenem

☐ Eravacycline

☐ Flucloxacillin

☐  Fluconazole

☐ Ganciclovir

☐ Gentamicin

☐ Imipenem

☐ Imipenem/relebactam

☐ Levofloxacin

☐ Linezolid

☐ Meropenem

☐ Meropenem/vaborbactam

☐ Metronidazole

☐ Micafungin

☐ Oritavancin

☐ Oxacillin

☐ Benzylpenicillin (Penicillin G) ☐ Piperacillin/tazobactam

☐ Refafungin

☐ Telavancin

☐ Teicoplanin

☐ Tigecycline,

☐ Tobramycin

☐ Trimethoprim/sulfamethoxazole

☐ Valganciclovir

☐ Vancomycin

☐ Other Click or tap here to enter text.*

*

1. *Which of the following antimicrobials do you administer as a bolus IV push or short infusion (not more than 1 hour) in your OPAT practice? Please select all that apply.

☐ Acyclovir

☐ Amikacin

☐ Amphotericin B

☐ Amoxicillin

☐ Ampicillin

☐ Ampicillin/Sulbactam

☐ Aztreonam

☐ Benzylpenicillin (Penicillin G)

☐ Caspofungin

☐ Cefazolin

☐ Cefiderocol

☐ Cefepime

☐ Cefoxitin

☐ Cefpodoxime

☐ Ceftazidime

☐ Ceftazidime/avibactam

☐ Ceftolozane/tazobactam

☐ Ceftriaxone

☐ Cephalexin

☐ Ciprofloxacin

☐ Clarithromycin

☐ Colistin

☐ Dalbavancin

☐ Daptomycin

☐ Doripenem

☐ Ertapenem

☐ Eravacycline

☐ Flucloxacillin

☐ Fluconazole

☐ Ganciclovir

☐ Gentamicin

☐ Imipenem

☐ Levofloxacin

☐ Linezolid

☐ Imipenem/relebactam

☐ Meropenem

☐ Meropenem/vaborbactam

☐ Metronidazole

☐ Micafungin

☐ Oritavancin

☐ Oxacillin

☐ Benzylpenicillin (Penicillin G)

☐ Piperacillin/tazobactam

☐ Refafungin

☐ Telavancin

☐ Teicoplanin

☐ Tigecycline,

☐ Tobramycin

☐ Trimethoprim/sulfamethoxazole

☐ Valganciclovir

☐ Vancomycin

☐ *Other Click or tap here to enter text.

1. *Do you perform therapeutic drug monitoring (TDM) for antimicrobials in the OPAT setting?

☐Yes ☐No

* If yes, please identify for which antimicrobial agent you perform TDM (Tick all that apply).

☐ Acyclovir

☐ Amikacin

☐ Amphotericin B

☐ Amoxicillin

☐ Ampicillin

☐ Ampicillin/Sulbactam

☐ Aztreonam

☐ Benzylpenicillin (Penicillin G)

☐ Caspofungin

☐ Cefazolin

☐ Cefiderocol

☐ Cefepime

☐ Cefoxitin

☐ Cefpodoxime

☐ Ceftazidime

☐ Ceftazidime/avibactam

☐ Ceftolozane/tazobactam

☐ Ceftriaxone

☐ Cephalexin

☐ Ciprofloxacin

☐ Clarithromycin

☐ Colistin

☐ Dalbavancin

☐ Daptomycin

☐ Doripenem

☐ Ertapenem

☐ Eravacycline

☐ Flucloxacillin

☐ Fluconazole

☐ Ganciclovir

☐ Gentamicin

☐ Imipenem

☐ Imipenem/relebactam

☐ Levofloxacin

☐ Linezolid

☐ Meropenem

☐ Meropenem/vaborbactam

☐ Metronidazole

☐ Micafungin

☐ Oritavancin

☐ Oxacillin

☐ Benzylpenicillin (Penicillin G)

☐ Piperacillin/tazobactam

☐ Refafungin

☐ Telavancin

☐ Teicoplanin

☐ Tigecycline,

☐ Tobramycin

☐ Trimethoprim/sulfamethoxazole

☐ Valganciclovir

☐ Vancomycin

☐ *Other Click or tap here to enter text.

1. *Do you ever use a loading dose when you initiate OPAT with continuous administration/infusion?

☐Yes ☐No

* If yes, please identify for which antimicrobial agent you would use a loading dose?

☐ Acyclovir

☐ Amikacin

☐ Amphotericin B

☐ Amoxicillin

☐ Ampicillin

☐ Ampicillin/Sulbactam

☐ Aztreonam

☐ Benzylpenicillin (Penicillin G)

☐ Caspofungin

☐ Cefazolin

☐ Cefiderocol

☐ Cefepime

☐ Cefoxitin

☐ Cefpodoxime

☐ Ceftazidime

☐ Ceftazidime/avibactam

☐ Ceftolozane/tazobactam

☐ Ceftriaxone

☐ Cephalexin

☐ Ciprofloxacin

☐ Clarithromycin

☐ Colistin

☐ Dalbavancin

☐ Daptomycin

☐ Doripenem

☐ Ertapenem

☐ Eravacycline

☐ Flucloxacillin

☐ Fluconazole

☐ Ganciclovir

☐ Gentamicin

☐ Imipenem

☐ Imipenem/relebactam

☐ Levofloxacin

☐ Linezolid

☐ Meropenem

☐ Meropenem/vaborbactam

☐ Metronidazole

☐ Micafungin

☐ Oritavancin

☐ Oxacillin

☐ Benzylpenicillin (Penicillin G)

☐ Piperacillin/tazobactam

☐ Refafungin

☐ Telavancin

☐ Teicoplanin

☐ Tigecycline

☐ Tobramycin

☐ Trimethoprim/sulfamethoxazole

☐ Valganciclovir

☐ Vancomycin

☐ *Other Click or tap here to enter text.

1. *Which dosing regimen do you use for meropenem in your OPAT practice?

☐2 g 24-hour infusion

☐2 g 12-hour infusion twice daily

☐4 g 24-hour infusion

☐3 g 12-hour infusion twice daily

☐ 3 g short infusion twice daily

☐6 g 24-hour infusion

☐1 or 2 g bolus infusion three times daily

☐ *Other. Click or tap here to enter text.

☐We don’t use meropenem in OPAT

1. *Which dosing regimen do you use for ceftazidime in your OPAT practice?

☐ 2g 8-hr infusion thrice daily

☐ 6g 24hrs infusion

☐ 2g 12hrs infusion twice daily

☐ 3g 12hrs infusion twice daily

☐ 4g 24 hours infusion

☐ 6g 24hrs infusion

☐ 2g 24hrs infusion

☐ *Other dosage regimen Click or tap here to enter text.

☐We don’t use ceftazidime in OPAT

*- Mandatory fields
